# Use and Outcomes of Acute Treatment Strategies in Patients with Severe Aortic Valve Stenosis

**DOI:** 10.5334/gh.1055

**Published:** 2021-12-27

**Authors:** Sven M. Piepenburg, Klaus Kaier, Christoph B. Olivier, Wolfgang Bothe, Timo Heidt, Markus Jäckel, Alexander Peikert, Dennis Wolf, Manfred Zehender, Christoph Bode, Daniel Dürschmied, Constantin von zur Mühlen, Peter Stachon

**Affiliations:** 1 Medical Center – University of Freiburg, University Heart Center, Department of Cardiology and Angiology I, Freiburg, DE; 2 Medical Center – University of Freiburg, Institute of Medical Biometry and Statistics, Freiburg, DE; 3 Center of Big Data Analysis in Cardiology (CeBAC), Medical Center – University of Freiburg, University Heart Center, Department of Cardiology and Angiology I, Freiburg, DE; 4 Medical Center – University of Freiburg, University Heart Center, Department of Cardiovascular Surgery, Freiburg, DE; 5 First Department of Medicine, University Medical Centre Mannheim (UMM), Faculty of Medicine Mannheim, University of Heidelberg, Mannheim, DE

**Keywords:** balloon valvuloplasty, transcatheter aortic valve replacement, surgical aortic valve replacement

## Abstract

**Background:**

This study aimed to evaluate the acute treatment of patients with severe aortic valve stenosis in Germany.

**Methods and Results:**

Three treatment strategies in 11,027 patients acutely admitted due to aortic valve stenosis were compared from 2014 until 2018 using German nationwide records: The annual number of transcatheter aortic valve replacement (TAVR) procedures (1,294 to 1,827) and balloon valvuloplasty (BV only) procedures (170 to 233) in patients acutely admitted increased, but surgical aortic valve replacement (SAVR) procedures decreased (426 to 316). In comparison to BV only patients (mean age 81.3; EuroSCORE 23.2) SAVR patients were younger and at lower logistic EuroSCORE (mean age 66.9; EuroSCORE 9.4). Patients treated with TAVR were at comparable age and operative risk (mean age 81.3; EuroSCORE 24.4) as those patients treated with BV only. Primary outcome was in-hospital mortality. Reimbursement was considered secondary outcome. After risk adjustment using multivariable logistic and linear regression analyses, SAVR (OR 0.26 [96%CI 0.16;0.45], p < 0.001) and TAVR (OR 0.38 [0.29;0.49], p < 0.001) were associated with lower risk for mortality compared to BV only. Compared to BV only, hospitalization costs of patients undergoing SAVR were reduced by €5,578 ([95%CI €8,023; €3,133], p < 0.001). TAVR procedures were associated with higher hospitalization costs than BV only (risk-adjusted difference €4,143 [€2,330; €5,926], p < 0.001).

**Conclusions:**

BV only was associated with a substantially increased risk of in-hospital mortality in acute patients. We conclude that a definitive aortic valve replacement should be preferred as primary treatment in patients with severe aortic valve stenosis causing an acute admission.

## Introduction

Severe aortic stenosis is common among elderly patients with a prevalence of 3.4% and associated with acute heart failure [1,2,3], cardiogenic shock and death [4]. In high-risk patients mortality without aortic valve replacement is around 50% within one year [5]. Due to the development of transcatheter aortic valve replacement (TAVR), more patients are nowadays eligible for aortic valve replacement [6]. TAVR interventions have been increasing continuously over the last years, with a similar in-hospital mortality compared to surgical aortic valve replacement (SAVR) after excluding emergencies and favourable long-time outcomes [7,8,9].

Initially only recommended for elderly and inoperable patients, rates of death, stroke and re-hospitalizations were seen to be lower for TAVR compared to SAVR in the PARTNER 3 trial of patients with low risk and a mean age of 73 years [8]. Nevertheless, SAVR remains an important alternative option with good long-term results for younger patients at low operative risk and in cases where TAVR is unfavourable due to anatomic reasons [10,11].

Patients with critical stenosis may present as an emergency with a beginning cardiogenic shock or multiple organ failure [12]. In these cases the operative risk for SAVR is increased and patients are often inoperable [13]. Accordingly, the first TAVR in men was performed by Alain Cribier in an emergency patient, who was rejected for SAVR [14]. Minimal invasive procedures, e. g. transcatheter aortic valve replacement (TAVR) and balloon valvuloplasty (BV) offer alternative treatment options in these unstable and endangered patients [15]. BV might be used as bridge-to-replacement or as destination therapy (BV only) [16]. However, BV only has been associated with high restenosis rates and increased mortality [5,17,18]. Therefore, the relevance of BV only as a destination therapy declines. Nevertheless, some BV only may be performed for palliative indications or in emergencies, where a TAVR or SAVR is not possible due to anatomic reasons or availability of TAVR or SAVR e.g. in certain smaller hospitals [19,20]. Emergency situations in particular make it difficult to decide on one of the three different treatments.

To our knowledge no registry studies evaluating larger numbers of symptomatic patients with emergency interventions such as TAVR, SAVR, or BV only have been performed yet. We therefore analysed clinical characteristics, comorbidities and outcomes of all three treatment options in acutely admitted patients undergoing interventions in a large nationwide cohort from Germany in order to provide more evidence for treatment of acute admitted cases with aortic valve stenosis.

## Materials and Methods

### Cohort definition

Since 2005, data on all hospitalizations in Germany have been available for scientific use via the Diagnosis Related Groups (DRG) statistics collected by the Research Data Center of the Federal Bureau of Statistics (DESTATIS). These data include diagnoses and procedures of nationwide in-hospital treatment of patients, reimbursed according to the DRG system. From this database we extracted data on all BV only, TAVR or SAVR procedures that were conducted among symptomatic patients with severe aortic valve stenosis, classified as acutely admitted admissions according to the DRG system (occasion of admission: emergency ‘aufn_anl N’). BV only, TAVR or SAVR procedures were defined including the respective procedures (OPS code 8837a0, 535a0, 53510) but excluding concomitant procedures (coronary artery bypass grafting, tricuspid valve or mitral valve replacement as defined by Reinöhl et al. [2015][6]). For the BV only group, a TAVR or SAVR procedure within the same hospital stay were excluded.

Our study did not involve direct access to data on individual patients by the investigators but only access to summary results provided by the Research Data Center. Therefore, approval by an ethics committee and informed consent were not required, in accordance with German law. All summary results were anonymized by DESTATIS. In practice, this means that any information allowing the drawing of conclusions regarding a single patient or a specific hospital is censored by DESTATIS to guarantee data protection. Especially the use of the anonymous and persistent ‘institute indicator of hospitals’ is restricted in order not to publish any information directly attributable to a single hospital.

### Outcome definitions

The primary outcome was in-hospital mortality. As secondary outcome total reimbursement was analysed. The used codes are described in our previous work [6,21].

### Statistical considerations

Continuous variables are reported as means ± standard deviations (SD) and frequencies were presented with percentages. Time trends were calculated using linear regression models.

In order to determine the impact of different procedures on the outcomes, multivariable logistic or linear regression analyses were carried out. A total of 21 baseline patient characteristics were included as potential confounders (all covariates listed in Table 1). In order to account for the correlation of error terms of patients treated in the same hospital, a random intercept was added at the centre level. All analyses were carried out using Stata 16.0 (StataCorp, College Station, Texas, USA).

**Table 1:** Baseline characteristics.

	Patients treated with SAVR (n = 1873)	Patients treated with TAVR (n = 8184)	Patients treated with balloon valvuloplasty (n = 970)	P- value SAVR vs. TAVR	P-value SAVR vs. BV	P-Value TAVR vs. BV

Age, years ± SD	66.9 ± 10.8	81.3 ± 6.6	81.3 ± 7.0	<0.001	<0.001	1.000
Women, %	30.8	50.2	46.8	<0.001	<0.001	0.041
In-hospital mortality, %	3.5	5.1	20.7	0.002	<0.001	<0.001
Reimbursement, mean ± SD	23.127€ ± 16.888€	34.781€ ± 11.096€	31.378€ ± 22.350€	<0.001	<0.001	<0.001
Cardiogenic shock, %	4.8	4.6	22.2	0.752	<0.001	<0.001
EuroSCORE	9.4 ± 7.5	24.4 ± 14.2	23.2 ± 13.9	<0.001	<0.001	0.013
NYHA class II, %	10.3	7.7	5.6	<0.001	<0.001	0.018
NYHA class III or IV, %	35.5	55.8	65.6	<0.001	<0.001	<0.001
Coronary artery disease, %	22.0	52.2	91.8	<0.001	<0.001	<0.001
Hypertension, %	57.2	62.2	53.2	<0.001	0.042	<0.001
Previous myocardial infarction <4 months, %	0.6%	1.9%	1.3%	<0.001	0.058	0.191
Previous myocardial infarction <1 year, %	0.2	0.8	0.9	0.007	0.007	0.629
previous myocardial infarction >1 y, %	2.5	5.5	4.2	<0.001	0.012	0.094
Previous CABG, %	2.0	11.2	5.9	<0.001	<0.001	<0.001
Previous cardiac surgery, %	5.3	17.2	10.3	<0.001	<0.001	<0.001
Atherosclerosis, %	4.6	12.1	13.8	<0.001	0.001	0.129
Carotid disease, %	4.6	6.3	6.2	0.004	0.068	0.862
COPD, %	11.1	14.7	11.6	<0.001	0.724	0.008
Pulmonary hypertension, %	13.6	25.8	23.5	<0.001	<0.001	0.115
Severe renal insufficiency (GFR <15 ml/min), %	1.8	3.4	4.4	<0.001	<0.001	0.106
Renal insufficiency (GFR <30 ml/min), %	1.8	6.3	7.8	<0.001	<0.001	0.065
Atrial fibrillation, %	41.0	50.7	47.3	<0.001	0.001	0.048
Diabetes mellitus, %	24.6	33.8	36.6	<0.001	<0.001	0.078

SAVR – surgical aortic valve replacement; TAVR – transcatheter aortic valve replacement; BV – balloon valvuloplasty; NYHA – New York Heart Association; CABG – coronary artery bypass graft; COPD – chronic obstructive pulmonary disease; GFR – glomerular filtration rate; SD – standard deviation.

### Patient and Public Involvement statement

The results were provided by the German Data Research Center. The aim of the collaboration of DESTATIS with public research institutes is to give the possibility to assess medical treatment in clinical practice in a nationwide cohort. Therefore, the collaboration is a tool for researchers to investigate clinical important problems, beyond that it serves as a quality control for public health.

## Results

### Prevalence of procedures for severe aortic valve stenosis in patients acutely admitted

We analysed characteristics and outcomes of 11,027 patients acutely admitted who underwent procedures in Germany for severe symptomatic aortic valve stenosis between 2014 and 2018. Over this time, 1,873 patients were treated with SAVR, 8,184 patients with TAVR and 970 patients with BV only (Table 1).

**Figure 1:**
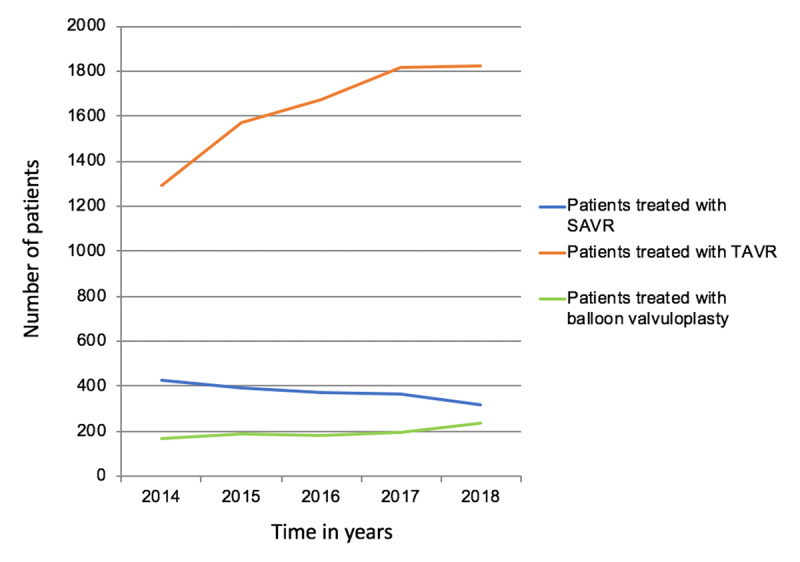
Procedures per year in patients with severe aortic valve stenosis acutely admitted: SAVR, surgical aortic valve replacement; TAVR, transcatheter aortic valve replacement.

Figure 1 reflects the number of emergency patients treated with SAVR, TAVR or BV only in Germany from 2014 until 2018. There was an increase in TAVR interventions (1,294 to 1,827, p = 0.014) and for BV only interventions (170 to 233, p = 0.054) per year. However, surgical aortic valve replacements decreased from 426 to 316 (p = 0.009).

### Baseline characteristics

Baseline characteristics are shown in Table 1. Patients treated with TAVR or BV only were substantially older than patients treated with SAVR (81.3 versus 66.9 years). They also had a higher EuroSCORE and more often fell into NYHA class III or IV than patients with SAVR. Nearly 50% of TAVR and BV only patients were women, in comparison to 31.2% of the SAVR group. TAVR patients had significantly more comorbidities than SAVR patients (see Table 1).

### Outcomes

Table 1 shows the outcomes of patients undergoing emergency treatment strategies for acute symptomatic aortic valve stenosis. Patients treated with BV only had the highest mortality rate (20.9%) while patients treated with TAVR were associated with the highest reimbursement (€34,781) without risk-adjustment.

**Figure 2:**
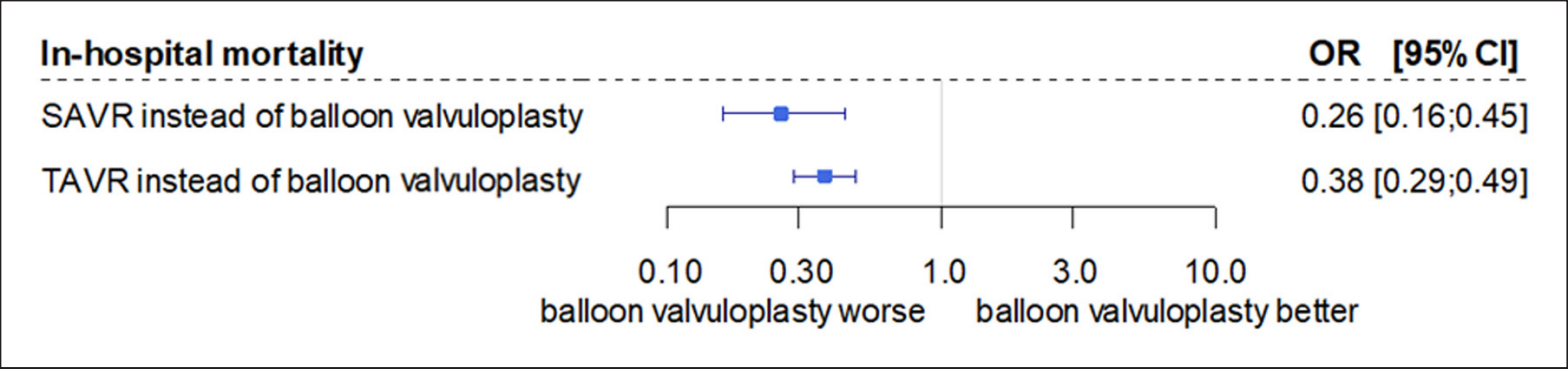
In-hospital mortality risk comparison of SAVR and TAVR versus balloon valvuloplasty OR, odds ratio (adjusted for all baseline patient characteristics according to Table 1); CI, confidence interval; SAVR, surgical aortic valve replacement; TAVR, transcatheter aortic valve replacement.

**Figure 3:**
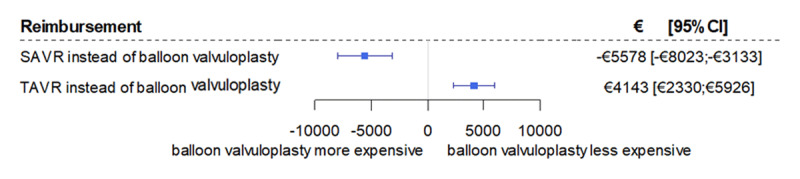
Reimbursement comparison of SAVR and TAVR versus balloon valvuloplasty OR, odds ratio (adjusted for all baseline patient characteristics according to Table 1); CI, confidence interval; SAVR, surgical aortic valve replacement; TAVR, transcatheter aortic valve replacement.

After risk adjustment, substantial differences were found for in-hospital mortality and reimbursement. In comparison to BV only, SAVR (OR 0.26 [96% CI 0.16; 0.45], p < 0.001) and TAVR (OR 0.38 [0.29; 0.49], p < 0.001) were associated substantially lower risk for in-hospital mortality (Figure 2). At the same time, the procedure-related increases in reimbursement are moderate. Compared to BV only, hospitalization costs of patients undergoing SAVR are reduced by €5,578 ([95% CI €8,023; €3,133], p < 0.001), despite the resource-intensive surgical procedure. TAVR procedures are associated with higher hospitalization costs (€4,143 [€2,330; €5,926], p < 0.001) (Figure 3).

## Discussion

To our knowledge, this is the first large registry study of acutely admitted patients undergoing emergency treatment strategies with SAVR, TAVR or BV only for severe aortic valve stenosis.

Our results expand the recently published study of Gaede et al. by comparing BV only therapy to TAVR and SAVR in patients with urgent need for treatment and analysing the costs of all three procedures [7]. BV only was associated with a substantially increased risk of in-hospital mortality. TAVR procedures for acutely admitted patients are increasing with similar outcome risks compared to SAVR and therefore promising for inoperable patients.

Patients with severe aortic valve stenosis who were admitted to the hospital acutely were mostly elderly patients at increased operative risk. This confirms the scientific consensus that even in emergency settings severe aortic valve stenosis is mainly a disease of elderly patients [22].

In accordance with several nationwide registries [23,24], we also observed a trend towards more TAVR than SAVR procedures, but with an additional reference to emergency admissions with severe aortic valve stenosis from 2014 until 2018. Interestingly, despite a widely described high complication rate of BV only [5,17,18], the number of BV only procedures increased even within the TAVR era in Germany. Of note, we included only cases into the BV only group, which did not receive TAVR or SAVR. The unfavourable in-hospital outcomes of BV only were confirmed in the present analysis.

Since reasons of treatment decisions are not documented, we can only speculate, why an aortic valve replacement was declined. Some BV only could have resulted from palliative indications and the increase of BV only could also be the result of increasing experience in transcatheter procedures [25], which even led to an increased use of similar techniques such as BV. Moreover, in some emergencies TAVR or SAVR may not have been possible due to anatomic reasons. Another cause may be restrictions in the availability of TAVR or SAVR. Hospitals without a department of cardiac surgery or low volume are not allowed to perform TAVR in Germany. In these smaller hospitals, BV only may be used as a rescue therapy [19,20]. However, even if some patients are referred for definitive replacement the mortality after BV only is still high.

Procedures for acutely admitted patients with symptomatic and severe aortic valve stenosis are challenging and Bongiovanni and his colleagues already reported a 30-day cardiovascular mortality rate of 23.8% for TAVR and 33.0% for BV [26]. In our own cohort, the mortality after SAVR was 3.5%, after TAVR 5.1%, and after BV only 20.7%. In comparison to both aortic valve replacement therapies, BV only patients had the worst outcomes in terms of in-hospital mortality even after risk adjustment. However, it is necessary to mention that these patients were sickest and there is likely a lot of residual confounding which cannot be completely covered by our registry study. A poor left ventricular function, advanced comorbidities and significant frailty could therefore lead to BV as a diagnostic tool to assess ‘therapeutic response’ instead of TAVR or SAVR, but also to symptom palliation in the context of cardiogenic shock [27]. Our results are in line with a report of Ben-Dor et al. who found a worse long-term survival in patients undergoing BV only compared to those who had BV to bridge for TAVR or SAVR [28]. High complication and restenosis rates have already been described for BV, but its method has improved and it still has a role as a bridging strategy to TAVR or SAVR [29]. Even though we are unable to tell if BV only patients might have benefited from bridging to valve replacement, we can provide evidence that BV only without definitive valve replacement is associated with high complication rates within a hospital stay. Therefore, if possible, a definitive aortic valve replacement should be planned within the hospital stay after BV, provided a life expectancy over one year according to current guidelines [10].

The shift towards TAVR is still associated with an increase of in-hospital costs [30]. The present study confirms that treatment costs were highest in the TAVR group in emergency settings. From an economic view BV only has no advantages: it has similar costs but significantly higher length of hospital stay and mortality compared to TAVR.

### Strengths and limitations

The strengths of this study include a large study population of acutely admitted patients with symptomatic severe aortic valve stenosis, first time comparison of clinical characteristics and outcome risk analyses according to the three treatment groups SAVR, TAVR or BV. Several limitations need to be considered. Analyses were performed in a registry study setting from a national database according to ICD and OPS codes. Important clinical factors of patients such as a decision to palliative care might therefore not have been considered. Furthermore, there was no follow-up to evaluate long-term outcomes of the three treatment groups. We examined BV only therapy and therefore cannot make any statement on its use in bridging to SAVR or TAVR.

## Conclusion

Patients acutely admitted with severe aortic valve stenosis undergoing BV are at increased risk of in-hospital mortality. They were also sicker than TAVR and SAVR patients which probably influenced our results. Thus, our findings support the concept of a definitive aortic valve replacement within the hospital stay whenever possible and indicated. Since outcomes of TAVR and SAVR were comparable in acutely admitted patients, an individual decision should be made in accordance to current guidelines.

## Figures and Tables

**Table:** 

BV	balloon valvuloplasty
DESTATIS	Research Data Center of the Federal Bureau of Statistics
DRG	Diagnosis Related Groups
LoS	Lengths of stay
SAVR	surgical aortic valve replacement
SD	standard deviations
TAVR	transcatheter aortic valve replacement
